# Increasing Equity Through Technology: A Comparison of Opinion Leader Identification Methods in Primary Care

**DOI:** 10.1177/00469580251370512

**Published:** 2025-09-09

**Authors:** Alan Kunz-Lomelin, Rebecca L. Mauldin, Thomas Valente

**Affiliations:** 1 Florida Atlantic University, FL, USA; 2 The University of Texas at Arlington, TX, USA; 3 University of Southern California, Los Angeles, CA, USA

**Keywords:** opinion leader, implementation science, diffusion of innovation, healthcare, health disparities, technology

## Abstract

Identifying low-cost implementation strategies to facilitate the uptake of technological innovations can help low-resource community clinics mitigate health disparities. Using a social network approach to identify organizational opinion leaders (OLs) can facilitate the adoption of innovations. To fill knowledge gaps related to alternative methods of identifying OLs, we identify and compare OLs in a low-resource community clinic using theoretically based techniques using Phi correlations and a binary logistic regression. Results showed that OLs identified through 3 out of 4 non-network identification methods (self-identification, positional, and staff selection) were significantly positively correlated with OLs identified using a social network approach. In addition, combining positional and staff selection methods was also found to be significantly associated with OLs identified using the social network approach. Implications for public health include the potential for non-network identification techniques to identify OLs to increase the uptake of technological innovations in low resource community clinics.

## Introduction

Low resource community clinics are well positioned to use technological innovations, such as emerging artificial intelligence (AI) technologies (eg, natural language processing, large language models, and predictive analytics), to mitigate health disparities because they are often the first point of contact for disadvantaged groups looking for support with health and social need.^1^ They are often tasked with helping mitigate disparities and increase healthcare access, which means that their work can magnify or mitigate inequities. Unfortunately, the challenges of implementation and uptake of technological innovations are often more difficult within low resource community clinics, which have limited resources compared to large or private health institutions.^2^

Limited uptake of innovative technologies can be detrimental, since some of these technologies (eg, AI use to facilitate documentation and diagnoses) have demonstrated their ability to reduce health disparities and address clinic challenges such as limited resources and staff shortages.^3,4^ Obstacles faced by these clinics during the uptake of technological innovations include difficulties using or understanding the benefits of the technology.^5,6^ These challenges could potentially be addressed through the identification of influential members within the network, “opinion leaders,” who can facilitate implementation efforts by providing advice and influencing their colleagues’ adoption of new technologies.^7-9^ Opinion leaders can help bring healthcare innovations by influencing the beliefs, attitudes, and behaviors of others within their network.^9-13^ For example, a review of 6 randomized clinical trials found that within the healthcare context, peer-identified opinion leaders have the potential to improve professional practice and patient outcomes.^11^

There is also ample evidence showing how the identification of opinion leaders can be useful for implementation in various settings and innovations.^14-16^ Past studies have found that the identification of opinion leaders in low resource settings is possible and can be effective in influencing the adoption of innovations and uptake of health behaviors.^10,17^ A qualitative study with community members and opinion leaders (identified through their positions of influence) in a Nigerian community found that opinion leader involvement helped increase awareness and uptake of various health behaviors focused on women and children.^10^ Another study found that establishing collaborations between peer-identified opinion leaders (identified through nominations) and providers could help increase adoption of recommended classroom practices for children with attention-deficit/hyperactivity disorder.^17^ These studies highlight 2 approaches that have the potential to be effective interventions for the identification of opinion leaders in low resource settings, and which can lead to greater adoption of innovations and health behaviors.

### Theoretical Framework for Comparing Opinion Leader Identification Techniques

There are several ways to identify opinion leaders. In the *sociometric*, or social network, approach, all or many members of a network are queried about their advice seeking, and individuals with the most nominations as advice-givers (ie, highest *indegree centrality*) are identified as opinion leaders.^9,12,18^ This approach maps the entire community and can provide high validity and reliability. When the early adopters of an innovation are also opinion leaders identified through the sociometric approach, it can accelerate diffusion of the innovation.^8^ However, early adopters are often on the periphery of the network with few connections to others.^19-21^ This results in slower adoption rates because it takes longer for the innovation to reach an opinion leader who can set the agenda for change and accelerate the diffusion to the rest of the network. The sociometric approach is significant because it is the only opinion leader identification technique that can empirically and directly identify network members with the highest connectedness or influence in a network. In addition, this approach not only reveals cohesion (direct advice-seeking relationships) within the network but also structural similarity (similar patterns of advice-giving relationships), which has also been found to be a stronger predictor of intervention adoption.

In their review of the techniques used to identify opinion leaders within an organization or community, Valente and Pumpuang argue that combining identification methods may lead to the identification of the most effective opinion leaders.^9^ However, resource limitations may restrict feasibility for using multiple approaches. In this case, prioritizing the *sociometric*, or social network, approach may be advisable. Unfortunately, it is not always feasible either due to resource limitations or uncertainty regarding the network boundary. Framed by the insights of Valente et al,^8,9^ our research seeks to build knowledge for the effective, yet feasible implementation of technological innovations in low-resource settings (eg, community clinics) by comparing various opinion leader identification methods against the sociometric approach and evaluating how different combinations of these methods may be associated with the identification of leaders through the sociometric approach.

### Comparing the Sociometric and Self-Identification Approaches

Identifying highly influential opinion leaders without having to conduct a whole network analysis may be possible. Historically in research, opinion leaders identified by the sociometric approach may also be identified by another approach, specifically the *self-identification approach*, which uses scores from a self-reported leadership scale to identify opinion leaders.^22-24^ These 2 approaches have been found to be significantly correlated in samples with farmers (*r* = .225) and vegetable growers (*r* = .408),^23,25,26^ physicians looking at the adoption of a new drug (*r* = .32),^22^ and Israeli and German adults (*r* = .54).^24^ However, studies conducted in a Scottish health care setting and a recent study in a South African village did not find these associations to be significant.^27,28^

### Current Study

Though most literature supports the idea that there is a significant association between leaders identified by the sociometric and self-identification approaches, inconsistent findings call for further research to understand in what contexts, settings, and populations this association exists. This research aims to build this evidence as well as fill other important knowledge gaps. First, most studies were carried out before 1991,^23,24,26^ which means that comparisons may not reflect the way social networks and relationships have changed over the years, as a result of emerging technologies in healthcare such as email, telehealth, videoconferencing, or social media. Furthermore, studies compare the sociometric approach to the self-identification approach, but no studies have looked at how other opinion leader identification methods may compare to the sociometric approach. Finally, the settings and networks from past studies^24-27^ differ from low resource community clinics in the United States. These gaps highlight the need to replicate and expand past literature to better understand the degree to which non-sociometric identification methods can identify influential leaders in the network.

This study’s long-term goal is to assist low-resource clinics with implementation efforts. It identifies influential opinion leaders through the sociometric approach and compares these to opinion leaders identified through non-sociometric techniques. The findings will expand and update the limited literature that compares opinion leader identification methods. This study poses 2 research questions related to opinion leaders within a low-resource community clinic system:

**Question 1:** To what extent are opinion leaders identified by the sociometric approach also identified by other opinion leader identification techniques, namely, self-identification, staff selection, self-selection, and positional approaches?
**Question 2:** What combinations of non-sociometric opinion leader identification techniques are significantly associated with opinion leaders identified through the sociometric approach.

### New Contribution

This article provides innovative insights into opinion leader identification approaches and the diffusion of innovations by comparing opinion leader identification techniques and combinations of these techniques, which have been understudied in the past. This study also takes a unique approach by looking at a low resource healthcare setting, which has implications for addressing health disparities through enhanced implementation of technological innovations. These findings offer organizations with low resources, staff shortages, or lower uptake of innovations different ways to identify influential opinion leaders, which can increase their access and adoption of technological innovations that may help low resource community clinics mitigate health disparities.

## Methods

The present study followed recommended guidelines from the EQUATOR network for cross sectional study data.^29^ This study utilized a cross-sectional survey conducted over a 2-month period (October 2023 to December 2023) using an online questionnaire among 121 employees within a network of 7 community care clinics in Texas. All aspects of this research were approved by the Institutional Review Board (IRB) at Baylor Scott and White Health (IRB # 023-297). Weekly consultation with research team members was carried out during the development, recruitment, and data analysis to identify potential sources of bias and make necessary adjustments or modifications to minimize bias. Participants were recruited from a group of Community Care (CC, pseudonym) clinics located in Texas. These clinics belonged to a large non-profit healthcare system supported by the Health Texas Provider Network. The goal of these clinics is to decrease health disparities by improving access and quality of healthcare services for underserved populations. The network of CC clinics is currently made up of 7 primary care sites that provide care to approximately 8500 patients. Members within the CC network often collaborate and refer patients to each other. In addition, these clinics offer a collaborative care model, which gives underserved patients access to appointments with primary care physicians, dieticians, pharmacists, social workers, community health workers, and other health professionals.

### Recruitment and Sample

Participants were eligible to participate if they were 18 years or older, an employee (not subcontractor) in a medical-related position at one of the CC clinics at the time of the study, and able to understand and read English. Additionally, they were excluded if they had a non-medical position (eg, custodial staff) or were employed at a different site that was not affiliated with 1 of the 7 CC clinics. The target sample size selected for the study was between 85 and 97 participants, which is equivalent to a 70% to 80% response rate. This sample size was selected because it meets the recommended threshold for the collection of whole network data of 70% to 80%.^30,31^ The 7 clinics had a total population of 121 employees (see Table 1) and 77% (n = 93) participated yielding a response rate that met this threshold.

**Table 1.** Clinic Employee Population (N = 121).

CC clinics	n	%
Clinic 1	25	21
Clinic 2	26	17
Clinic 3	22	14
Clinic 4	18	15
Clinic 5	14	11
Clinic 6	13	13
Clinic 7	5	4
More than 1 clinic^a^	21	22
Total	121	100

a Employees with positions of leadership or who have more than 1 home clinic.

A link to the online questionnaire was sent through email to every employee at the CC clinics. Additional recruitment strategies included placing flyers in breakrooms, attending staff meetings, and delivering envelopes with survey links during staff meetings and clinic visits.

### Data Collection and Measures

Upon completion of an online informed consent, participants were directed to an online questionnaire, which took up to 30 min to complete. Participants were eligible to receive 1 of 7 randomly awarded $50 gift cards. The online questionnaire included demographic questions, a group of opinion leadership identification items, and a validated Opinion Leadership Scale.^32^

#### Demographic Variables

Demographic items were measured using categorical multiple-choice questions except for years employed, age, and role. Years employed was calculated by asking participants to select the date when they were hired at their current position and subtracting this from the date of enrollment in the study. Age and role were open-ended items. Physicians, Physician Assistants (PA), and Nurse Practitioners (NP) were combined into a single “Medical Provider” role category. Licensed master social workers (LMSW) and licensed clinical social workers (LCSW) were combined into “Behavioral Health Provider.”

#### Opinion Leader Identification Variables

Participants also completed items for 5 opinion leader identification measures that used the sociometric, staff selection, self-selection, positional, and self-identification approaches. There are 5 other opinion leader identification techniques identified by Valente and Pumpuang not measured in this study.^9^ The celebrity approach was excluded due to lack of access to celebrities or knowledge about who may be considered a celebrity within the clinic. The judge’s ratings approach was excluded due to limited resources and insufficient data about the organization to identify knowledgeable community members. The expert identification approach was excluded due to lack of access to a trained ethnographer and the complexity of conducting observations in a clinical setting without interfering with clinic workflow. Finally, the snowball and sample sociometric approaches were excluded because the sociometric approach encompasses these sampling approaches by collecting data from the entire population.

When identifying opinion leaders, it is recommended to select the people who score in the top 10% to 15% of cases when the approach being used entails a score or count.^9,33^ In this study, we aimed for a cutoff of the top 10% of cases. However, because there were multiple employees with the same values of scores, using a strict 10% cutoff would have excluded some employees with the same scores from being identified as an opinion leader. Consequently, we relaxed the standard to include anyone who scored the same or higher as the 90th percentile case. Using this approach to identifying opinion leaders, 12% to 13% of employees were identified as opinion leaders for each of the 4 identification approaches included in this study that used scores or counts (ie, sociometric approach, staff selected, self-selected, and self-identified). These percentages fall within the recommended range of the top 10% to 15% of cases.^9,33^ There was only 1 variable (ie, self-identified opinion leaders, described below) in which a different cutoff point would have split the sample in a manner to achieve the recommended 10% to 15% range. To ensure our choice of cutoff did not affect our results, we conducted sensitivity analyses and found using this alternate cutoff point did not substantively change our findings.

##### Sociometric Items

Two sociometric items were asked and respondents were presented with a roster of all employees in the CC network to provide their responses (organized by clinic). The questions were “who do you go to for advice when you face a challenge at work” and “who comes to you for advice when you face a challenge at work.” In addition to selecting names, participants could select “no one from this clinic” or “prefer not to answer for this clinic.”

Responses from the advice seeking item were used to construct an advice seeking network such that tie *x_ij_* = 1 if *i* reported seeking advice from *j* and *x_ij_* = 0 if *i* did not seek advice from *j*. Missing data were imputed using the advice-giving response such that *x*_ij_ = 1 if *j* reported giving advice to *i* and *x_ij_* = 0 if *j* did not give advice to *i*. If data were missing for both *i*’s advice seeking from *j*, and *j*’s advice giving to *i*, no value was imputed for the advice seeking tie *x_ij_*.

The sociometric opinion leader variable was calculated using indegree centrality in the advice seeking network. A continuous count of the number of incoming ties in the advice seeking network (ie, indegree centrality) for each employee (with a range from 0 to 120) was first calculated in UCINET.^34^ Indegree centrality is often understood as a measure of popularity.^33,35,36^ Although there are dozens of centrality metrics that could potentially be used to identify opinion leaders in a network (see eg, Scoch’s (n.d.) periodic table of centrality measures),^37^ indegree centrality is the most commonly used^11,12^ and is a metric that is easy to calculate without specialized expertise in social network analysis, increasing its accessibility to low resource community clinics.

For the sociometric opinion leader variable, the 90th percentile case had an indegree of 17, so the cutoff score for operationalizing an opinion leader using the sociometric approach was anyone with indegree of 17 or higher. This resulted in the top 13% (n = 16) of employees, with indegree scores ranging from 17 to 35, being identified as sociometric opinion leaders. Sociometric opinion leaders were assigned a value of 1 = *yes* for sociometric opinion leaders meeting the threshold, while the remaining participants (87%) were assigned a value of 0 = *no* for the variable.

##### Staff-Selected Opinion Leaders

 The staff selection variable was based on an item that asked participants, “Who do you consider to be a leader in the area of technological innovations within your clinic and/or organization? (select as many as apply)” using the clinic roster. The 90th percentile case received 6 nominations from the participants, so a score of 6 or above was used to operationalize staff-selected opinion leaders. Fifteen employees (12%) met this threshold, receiving from 6 to 13 nominations, and were classified as staff selected opinion leaders. This variable was coded as 1 = *staff selected opinion leader* and 0 = *not a staff selected opinion leader*.

##### Self-Selected Opinion Leaders

 Self-selection is operationalized in the literature as those who respond positively to being asked if they would be willing to act as an OL within their organization.^9^ For this study self-selected opinion leaders were identified using scores on a single item asking, “How interested would you be in volunteering as a leader in the deployment, training, and/or implementation of innovations in your clinic or other CC clinics?,” which is similar to the way it has been measured in past literature.^9^ This item used a five-point Likert type rating scale ranging from 1 (very unlikely) to 5 (very likely). The self-selected opinion leader variable was a dichotomous variable calculated as 1 = *yes* for those who reported that they were “very likely” to volunteer to be a leader in the implementation of technological innovations (n = 16, 13%) and 0 = *no* for all others.

##### Self-Identification Opinion Leaders

In the literature, self-identified leaders are those who respond positively on a composite scale consisting of items asking them to rate how often they engage in activities that represent opinion leader behavior.^9^ In the current study, self-identification opinion leaders were assessed with a modified version of a leadership scale that has been used to measure self-reported opinion leadership among physicians.^22^ The Opinion Leadership Scale (OLS) in Iyengar et al’s study has not been validated itself; however, this scale is a modified version of a leadership scale that has been validated in the field of marketing.^22,32,38^ This scale contains 6 Likert-type questions with scores ranging from 1 to 7 for each item. The scores for each item are added to provide an overall leadership score, which can range between 6 and 42, with higher scores representing higher degrees of self-identified leadership. This study adapted Iyengar et al’s scale to ask clinic staff about the likelihood and frequency of interactions between the clinic employees and their co-workers around issues related to innovations at their clinics.^22^ The leadership scores were then dichotomized to create the self-identification opinion leader variable. Employees with the top 13% (n = 16) of scores (ranging from 33 to 40) were assigned a value of “1,” while the rest were assigned a value of “0.” For the self-identification approach, 12% of the data were missing and excluded from the analysis.

##### Positional Opinion Leaders

The positional opinion leader variable was measured using a categorical open-ended question that asked participants what their position or job title was within their organization. A dichotomous variable was created where “1” represented employees with positions of leadership within the CC Network (ie, managers, supervisors, directors, n = 24, 19%) and “0” employees without positions of leadership.

### Statistical Analysis

The descriptive and inferential statistics for this study were calculated using packages within R.^39^ The inferential statistics utilized a significance alpha of 0.05, and a correlation analysis using the Phi correlation coefficient^40^ was used to indicate the correspondence between opinion leader identification methods.

**Figure 1.**
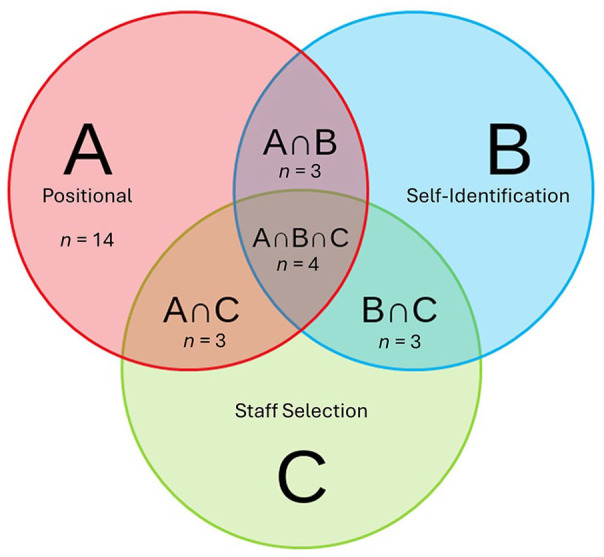
Venn diagram of non-sociometric opinion leader identification techniques and their overlap for a regression analysis.

In addition, a binary logistic regression was carried out where sociometric opinion leader status (yes/no) was regressed on each person’s opinion leader status as identified via the other opinion leader identification methods to determine correlation of the methods. Specifically, being a sociometric opinion leader was regressed on opinion leaders identified by the (A) positional, (B) self-identification, and (C) staff selection approaches. The Venn diagram in Figure 1 illustrates the overlap in the 3 non-sociometric techniques. The combination of all 3 methods (A ∩ B ∩ C) was excluded from the model to avoid multicollinearity.

For the regression analysis, positional and staff selection remained dichotomous variables (Y/N), while self-identification was continuous variable based on participant scores on the leadership scale. In addition, the A ∩ B (positional and self-identification) combination was dichotomized and received a “1” if a participant was identified as an opinion leader by only these 2 approaches. Combination A ∩ C (positional and staff selection) was calculated by multiplying the dichotomous positional value (Y/N) and the number of nominations received (staff selection). Finally, B ∩ C (self-selection and staff selection) was calculated by multiplying opinion leadership scores (self-identification) and nominations (staff selection).

## Results

Out of 121 participants, 93 completed the questionnaires, the remaining participants declined participation or chose not to participate by not going to the online survey. The sample (n = 93) was primarily female (90%), Hispanic (64%) and having at least some college education (see Table 2). Participants average age was 38 years, average length of employment at their current position was 6 years, average leadership score was 22, and mode volunteer score was 4.

**Table 2.** Demographics of the Sample of Employees Recruited for the Study (N = 93).

Variable	n	%	M	SD
Gender				
Male	9	9.7		
Female	84	90.3		
Race/Ethnicity				
White	12	13.2		
Hispanic/Latino(a)	58	63.7		
Black/AA	10	11.0		
Multiracial	2	2.2		
Other	9	9.7		
Education				
HS or GED	12	13.2		
Some college	28	30.1		
College graduate or higher	39.8	17.2		
Continuous variables				
Age	93		37.8	10.4
Years employed	93		5.9	4.6
Leadership Score	93		21.9	9.5
Volunteer Score	93		3.6	1.0

Additionally, there were diverse roles that participants had within the clinics (see Table 3) and a tendency for clinics to have a similar number of employees working within them (19-32), except for 1 clinic where only 7 employees reported working there. Participant roles within the clinics were diverse with leadership positions (13%) and medical assistants (20%) being the most common.

**Table 3.** Clinic Roles among the Sample of Employees Recruited for the Study (N = 93).

Variable	n	%
Role		
Manager/Supervisor/Director	17	18.3
Medical assistant	19	20.4
Community health worker	12	12.9
Pharmacist/Pharmacy Tech	12	12.9
Medical provider	11	11.8
Patient services specialist	11	11.8
Behavioral health provider	6	6.5
Other	5	5.4

### Identifying and Comparing Opinion Leaders

The strongest correlations (see Table 4) were between the following opinion leader identification approaches: sociometric and positional (*Ф* = 0.444, *P* < .001), sociometric and staff selection (*Ф* = 0.469, *P* < .001), and staff selection and self-identification (*Ф* = 0.406, *P* < .001). In addition, weaker but significant correlations were found between the following approaches: positional and staff selection (*Ф* = 0.256, *P* = .004), positional and self-identification (*Ф* = 0.301, *P* = .006), and sociometric and self-identification (*Ф* = 0.231, *P* = .036). Interestingly, the self-selection (ie, volunteer) approach was not significantly correlated with any of the other approaches.

**Table 4.** Correlation Matrix Showing Phi Correlation Coefficients for Opinion Leader Identification Methods.

Opinion Leader Identification Variable	1	2	3	4
1.Network/Sociometric	—			
2.Positional	.444***	—		
3.Staff selection	.469***	.256**	—	
4.Self-identification	.231*	.301**	.406***	—
5.Self-selection (volunteer)	.081	.096	.080	.096

* *P* < .05. **P < .01. ***P < .001.

**Figure 2.**
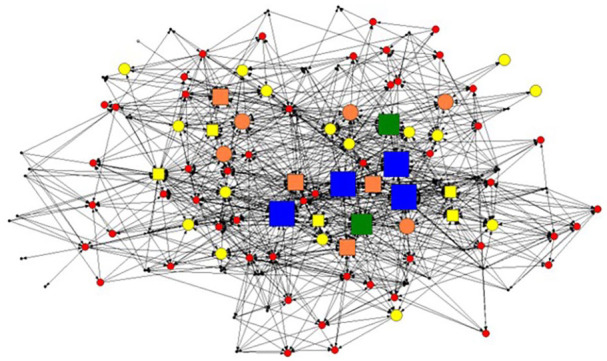
Advice seeking network and identified opinion leaders among employees of a community clinic network (N = 121). *Note*: Polygons represent employees; lines represent an advice-seeking tie; arrows point to the employee from whom advice is sought. Symbols for employees are coded by size, color, and shape to represent whether the employee was identified as an opinion leader, the number of identification methods through which they were identified as an opinion leader, and whether they were identified through the sociometric approach. Larger symbols indicate the employee was identified by more identification methods. Color also represents the number of methods through which an employee was identified as an opinion leader: Black = 0, Red = 1, Yellow = 2, Orange = 3, Green = 4, Blue = 5. In addition, nodes that are squares represent members in the network who were identified by the sociometric approach, while circles were not.

A visual representation of the significant correlations found between the sociometric approach and non-sociometric approaches (ie, positional, staff selection, and self-identification) can be found in Figure 2, where bigger shapes represent identification of opinion leaders through more approaches. The largest shapes in the network are located toward the center, signifying that opinion leaders identified through the sociometric approach (those with the most connections) may have a tendency to also be identified by other opinion leader identification methods.

Finally, the overall model for the regression was statistically significant χ² (6) = 30.97, *P* < .001, and explained approximately 71% of the variance (Nagelkerke *R²* = .71). Table 5 shows that the only combination of opinion leader identification approaches statistically significantly associated with the sociometric approach was the positional and staff selection combination (OR = 2.17, 95% CI [1.27-6.76]. *P* < .04).

**Table 5.** Regression of Combinations of Opinion Leader Identification Approaches to the Sociometric Approach.

Predictor	B	OR	95% CI
Positional	−0.71	0.49	0.00-7.24
Self-identification	−0.01	0.99	0.86-1.13
Staff selection	2.41	11.17	0.89-163.32
Positional + self-identification	−0.20	0.82	0.00-42.00
Positional + staff selection	0.77*	2.17	1.27-6.76
Self-identification + staff selection	0.01	1.01	0.99-1.02

* *P* < .05.

## Discussion

The results of this study reveal the degree to which non-sociometric opinion leader identification techniques correlate with the sociometric approach and each other. Results add to the current knowledge around diffusion of innovation strategies in low resource community clinics, whose work focuses on increasing health equity and mitigating health disparities. Findings from this study offer insights that may enable low resource community clinics to incorporate technological innovations into their workspace in an efficient way, while mitigating the barriers of limited resources and lower uptake of innovations often found in this setting.^2^ These findings may equip low resource clinics with insights they can use to enhance the implementation of technological innovations and as a result give them access to innovative tools to continue mitigating health disparities.

### Comparing Opinion Leader Identification Methods

This study offers a more recent comparison of opinion leader identification approaches since past comparison studies had been done before 2011 and most before 1991.^23-26^ Furthermore, this study provides findings within the context of a healthcare setting in the United States, while a lot of past research had been done on non-clinical settings^25,26^ and countries other than the United States.^24,27^

Findings show significant correlations between the sociometric opinion leader identification approach and 3 other opinion leader identification approaches. The finding of a significant correlation (*Ф* = .231, *P* = .036) between the sociometric approach and the self-identification approach is consistent with past studies which have found moderate to strong correlations between them.^22,25,27^ However, findings from the current study help fill gaps in the literature by revealing significant correlations between the sociometric approach and other opinion leader identification approaches that have been understudied in the past (ie, positional and staff selection), which shows the potential of these approaches to identify influential opinion leaders in the network without the need to use a sociometric approach. However, the effect size for these significant correlations show a moderate (Φ ≈ 0.4) effect, and it remains unclear if the non-network approaches would adequately identify the appropriate members to effect change. Despite that, non-sociometric approaches show potential to identify sociometric opinion leaders when combined with other methods, as evidenced by the significant association found between the combination of the positional and staff selection approaches and the identification of opinion leaders through the sociometric approach.

### Practice Implications

The findings from this study have important implications for healthcare practice. The study’s findings show that clinic administrators have multiple implementation strategies they can follow to increase the uptake of technological innovations. Clinic administrators can choose to utilize approaches that require the development and administration of a questionnaire to identify and recruit influential opinion leaders in the network (ie, sociometric, self-identification, and staff selection). However, low resource community clinics may not have the time and resources needed to carry out a network wide data collection. Fortunately, findings from this study suggest the positional and staff selection approaches may provide clinics with a way to reap the benefits of opinion leader identification while mitigating the challenge of having to create and distribute a questionnaire throughout the network. Findings also suggest clinic administrators may choose to utilize a combination these approaches, since this combination has been found to be associated with the identification of opinion leaders who have high centrality within the network.

The positional approach allows clinics to instantly identify opinion leaders in their network without the need to invest time and resources in data collection, since the organization already has familiarity with the roles of their employees and who holds positions of leadership. The positional approach is not only time and cost efficient, but it has also demonstrated its potential to help identify influential opinion leaders, as evidenced by its significant correlation to the sociometric approach found in this study (*Ф* = .444, *P* < .001). Similarly, staff selection is a low-cost solution that identifies trusted network members through peer nominations, and which has also been found to be correlated to the sociometric approach (*Ф* = 0.469, *P* < .001). It is worth noting that other approaches may have benefits that the positional and staff selection approaches may not have, such as evidence of influence (sociometric) or leadership experience (self-identification). Clinic administration may choose one of these approaches when it is feasible or when they would like to prioritize one of these characteristics, but if they would like to prioritize time and cost efficiency while still having the potential to identify influential members in the network then the positional, staff selection, or a combination of both approaches may provide the best alternative.

### Policy Implications

This study’s findings also highlight policy implications that enhance our understanding of the implementation of technological innovations in healthcare. Since non-sociometric approaches (ie, positional and staff selection) were found to have the potential to identify influential members in the network (as evidenced by significant correlates of the opinion leader’s identified by the sociometric approach), low resource community clinics may benefit from finding ways to integrate these approaches into existing workflows. For example, healthcare systems often have annual reviews or yearly questionnaires that employees are required to complete as part of their employment within the healthcare system. Policies that incorporate opinion leader identification items or scales to these questionnaires may help low resource community clinics collect opinion leadership information annually without the need to invest additional time and resources collecting this data. The annual questionnaire can ask employees to nominate leaders for the implementation of technological innovations (staff selection approach) and to complete a leadership scale (self-identification approach). Adding these to a pre-existing questionnaire allows the organization to systematically collect up to date opinion leadership data without added burden to the clinics. In addition, turnovers, promotions, and retirements can change the networks and structure of the clinic. However, by identifying opinion leaders annually through this approach, organizations can stay up to date with who are opinion leaders in their network. By creating policies that make opinion leader identification a standard practice they can expedite the implementation process. Since opinion leaders will be already identified, low resource community clinics will be able to move to the next step of the implementation process and in doing this save additional time and resources.

### Limitations

There are limitations to consider when interpreting the findings of this study. First, the study was carried out in a unique network of 7 clinics and results and may not be generalizable to all healthcare contexts. In addition, there were limitations around getting an accurate roster of the network. The network members were constantly shifting due to turnovers, new hires, retirements, transfers, or employees quitting their positions. Recruitment was carried out over a 2-month period and as a result it was difficult to obtain an accurate CC roster. Furthermore, the questionnaires in this study were not pilot tested or validated, which may affect their validity. Given the clinical context of the study, respondents may have interpreted the self-selection question as a request for actual volunteers rather than purely a research question and may have been hesitant to express interest in being an opinion leader due to the perceived time or workload burdens. Future studies may consider rewording the question to say, “Would you feel comfortable providing expert opinions when asked by colleagues or sharing the latest practice guidelines,” which would more accurately identify individuals who see themselves as informal OLs or self-selected OLs without implying additional obligations.

Future studies must consider these limitations and target questions that may have been left unanswered. There are different types of technological innovations that can be utilized in primary care, future studies may compare these different technologies, assess their presence or absence in low resource community clinics, and their impact on health disparities.

### Conclusion

Several conclusions can be drawn from the findings in this study. Overall, opinion leader identification methods appear to be significantly associated with one another. Specifically, the positional and staff selection approaches appear to be significantly associated with the identification of opinion leaders through the sociometric approach, highlighting their potential to identify influential opinion leaders without the need to carry out a whole network analysis. Low resource clinics may be able to increase their uptake of technological innovations by utilizing these approaches during implementation efforts. These approaches can help these clinics overcome the barriers around the uptake of these innovations, while allowing them to identify opinion leaders that can be early adopters. However, the role of opinion leaders only constitutes an initial step in a multi-phase process, clinics must customize their implementation approaches by considering the type of technology that will be implemented, the degree to which this technology has been successfully implemented in similar settings, and the degree to which it may mitigate disparities.

The findings of the current study have added to the knowledge base for low resource community clinics for improving their implementation strategies and increasing their uptake of innovations. It is the hope that this increased uptake will grant low resource community clinics access to innovative technologies that they had not had access to in the past, but that will better equip them to continue mitigating health disparities through their work with underrepresented or marginalized groups.

## Supplemental Material

sj-docx-1-inq-10.1177_00469580251370512 – Supplemental material for Increasing Equity Through Technology: A Comparison of Opinion Leader Identification Methods in Primary Care Supplemental material, sj-docx-1-inq-10.1177_00469580251370512 for Increasing Equity Through Technology: A Comparison of Opinion Leader Identification Methods in Primary Care by Alan Kunz-Lomelin, Rebecca L. Mauldin and Thomas Valente in INQUIRY: The Journal of Health Care Organization, Provision, and Financing

sj-docx-2-inq-10.1177_00469580251370512 – Supplemental material for Increasing Equity Through Technology: A Comparison of Opinion Leader Identification Methods in Primary Care Supplemental material, sj-docx-2-inq-10.1177_00469580251370512 for Increasing Equity Through Technology: A Comparison of Opinion Leader Identification Methods in Primary Care by Alan Kunz-Lomelin, Rebecca L. Mauldin and Thomas Valente in INQUIRY: The Journal of Health Care Organization, Provision, and Financing
