# Supplementary material for: Increasing Equity Through Technology: A Comparison of Opinion Leader Identification Methods in Primary Care
Source: Inquiry. 2025 Sep 9;62:00469580251370512. doi: 10.1177/00469580251370512 (PMC12420978; doi:10.1177/00469580251370512)
Supplement: sj-docx-1-inq-10.1177_00469580251370512 – Supplemental material for Increasing Equity Through Technology: A Comparison of Opinion Leader Identification Methods in Primary Care [file sj-docx-1-inq-10.1177_00469580251370512.docx]

**Opinion Leader Identification Questionnaire**

**Directions:** You will be asked about the people who you seek advice from or who you give advice to within your Baylor Community Care clinic and other Baylor Community Care clinics. For each question, you will receive a roster of all the employees currently working at each clinic, please select the employees that appropriately describe the individuals that each question is asking about. ***Please note, roster is not included to maintain confidentiality**

- **Question #1:** At the BCC clinics, who do you go to for advice when you face a challenge at work? (Select all that apply)

[SOCIOMETRIC #1]

**[Participants have the option of selection one of these two choices for each clinic and question]**

- No one from this clinic
- Prefer not to answer for this clinic
- **Question #2:** At the BCC clinics, who comes to you for advice when they face a challenge at work? (Select all that apply)

[SOCIOMETRIC #2]

**[Participants have the option of selection one of these two choices for each clinic and question]**

- No one from this clinic
- Prefer not to answer for this clinic
- **Question #3:** Who do you consider to be a leader in the area of technological innovations within your clinic and/or organization? (Select all that apply)

[STAFF SELECTION]

- **Question #4:** If BSWH asked for volunteers to have a leadership role during the implementation of innovations within your clinic, how likely would you be to volunteer to be a leader in the deployment, training, and/or implementation of this?

[SELF-SELECTION]

1. Very unlikely 1. Unlikely 2. Neither Likely nor Unlikely 3. Likely 4. Very likely

- **Question #5:** What is your position or job title at BSWH? ___________________________________________

[POSITIONAL]
