# Supplementary material for: Increasing Equity Through Technology: A Comparison of Opinion Leader Identification Methods in Primary Care
Source: Inquiry. 2025 Sep 9;62:00469580251370512. doi: 10.1177/00469580251370512 (PMC12420978; doi:10.1177/00469580251370512)
Supplement: sj-docx-2-inq-10.1177_00469580251370512 – Supplemental material for Increasing Equity Through Technology: A Comparison of Opinion Leader Identification Methods in Primary Care [file sj-docx-2-inq-10.1177_00469580251370512.docx]

**Opinion Leadership Scale - Adaptation**

| In general, how often do you talk to others about innovations at your clinic or at any BCC clinic? |
| --- |
| Never Very Often  1 2 3 4 5 6 7 |
| When you talk to your colleagues about innovations at your clinic or at any BCC clinic do you: |
| Offer very little information Offer a great deal of information  1 2 3 4 5 6 7 |
| During the past year, how many BCC clinic employees have you instructed about ways to utilize an innovation introduced into your clinic or any BCC clinic? |
| Instructed no one Instructed multiple clinic employees  1 2 3 4 5 6 7 |
| Compared to your circle of colleagues, how likely are you to be asked about innovations at your clinic or any BCC clinic? |
| Not at all likely Very likely  to be asked to be asked  1 2 3 4 5 6 7 |
| In discussions of innovations at your clinic or at any BCC clinics, which of the following happens most often? |
| Your colleagues tell you about You tell your colleagues about  innovations innovations  1 2 3 4 5 6 7 |
| In general, when you think about your professional interactions with colleagues, are you: |
| Not used as a Often used as a  source of advice source of advice  1 2 3 4 5 6 7 |
